# What's in a disease like non‐alcoholic fatty liver disease—East *versus* West?

**DOI:** 10.1002/jgh3.12790

**Published:** 2022-08-02

**Authors:** Yock Young Dan

**Affiliations:** ^1^ National University Singapore Yong Loo Lin School of Medicine Singapore Singapore

Non‐alcoholic fatty liver disease (NAFLD) or its nemesis, metabolic associated fatty liver disease (MAFLD), is projected to become a worldwide epidemic, in association with the exponential rise in obesity and metabolic disease.^1^ While there is no argument on the histopathological criteria of NAFLD or Non alcoholic steatohepatitis (NASH) on gold standard liver biopsy, the understanding of fatty liver disease has evolved from a liver problem to a systemic condition where the fatty liver is the liver manifestation of a metabolic dysregulation.

Part of the challenge in managing NAFLD is the need to address the bigger problem of metabolic inflammation, but a practical problem exists. There is no good scientific way of defining metabolic status in the context where fatty liver arises. The Adult Treatment panel III (ATP‐III) criteria define metabolic syndrome as a group of conditions that together raise your risk of coronary heart disease, diabetes, stroke, and other serious health problems. Empirically, it is diagnosed when there are three out of five associated clinical diseases occurring together.^2^ The MAFLD consensus attempt to patch this gap by defining the disease based on histological, imaging, or blood biomarker evidence of hepatic steatosis, in addition to the presence of at least one of the following: obesity, type 2 diabetes mellitus, or metabolic dysregulation. The latter in turn requires at least two criteria based on waist circumference, pre‐diabetes, hypertension, hyperlipidemia, insulin resistance, or elevated high sensitivity C‐reactive protein.^3^

There are three unmet gaps in these approaches. (i) The criteria of MAFLD, though derived in consensus, are still empirically based and do not allow quantitative appreciation of accumulative metabolic risk. (ii) Waist circumference and obesity are but surrogate markers and do not take into account the more complex basis of ectopic fat deposition, lipotoxicity, and sarcopenia, features that are emerging as key pathological basis of insulin resistance driving metabolic complications.^4^ (iii) Differences between population subgroups, stereotypically and simplistically classified as East *versus* West, require different subclassification of body mass index (BMI) and call into question whether these consensus criteria can be universally applied.^5^ One good example is the entity of lean NAFLD, which has been described to be more common in Asia based on the observation that Asians develop metabolic complications at lower levels of BMI compared with their Western counterpart.^6^

Many East vs. West epidemiological studies of NAFLD have been reported. In this issue, Zhang *et al*.^7^ compared a prospective cohort of 160 patients who presented with NAFLD at the University of Michigan Health System in the United States and Peking University Health Sciences Centre in China and looked at the prevalence of metabolic syndrome as well as fibroscan measurements of hepatic steatosis and fibrosis. The quantity and quality of body fat and muscles were measured with computer tomography scan.

Overall, American patients had higher indices of liver steatosis and fibrosis, were correspondingly older, had higher prevalence of metabolic syndrome, and had more fat in liver, visceral, subcutaneous, and muscle compartments than Chinese patients. Even after matching for age, BMI category, and gender, Chinese patients have less advanced fibrosis, lower visceral fat density, and less subcutaneous fat area (SFA). The prevalence of metabolic syndrome was not statistically different. To explain the discrepancy, the authors surmised that Chinese patients had milder NAFLD due to exposure to shorter years of obesity, evoking the view that NAFLD in Asian community is a younger disease compared with the West. The severity of NAFLD, in both cohorts, however, is correlated with metabolic syndrome, which also manifests in more visceral, subcutaneous, and muscular fat.

Is there a real difference in NAFLD/MAFLD between the East and West? Asians have been reported to have higher visceral fat or fraction for the same BMI,^8^ but it is difficult to draw conclusions on NAFLD disease pattern as referral patterns, genetic makeup, and local lifestyle make direct comparisons difficult. Without a gold standard reference for even defining metabolic risk, matching for comparison is at best arbitrary. To truly understand interethnic differences in disease epidemiology, large population cohorts will need to be deeply and similarly characterized for meaningful comparison and followed up for longitudinal outcomes. As interesting as ethnic differences are, it is important to remember that beyond comparison of the severity of NAFLD/MAFLD or body composition, it is ultimately the ability to prognosticate patients based on comprehensive ethnic, genetic, and metabolic profiling that will rewrite the paradigm of the disease and allow us to focus screening and interventive efforts on those at highest risk.
